# Landmark Sequence Learning from Real-World Route Navigation and the Impact of Navigation Aid Visualisation Style

**DOI:** 10.5334/joc.307

**Published:** 2023-07-12

**Authors:** Christopher Hilton, Armand Kapaj, Sara I. Fabrikant

**Affiliations:** 1 Geographic Information Visualization & Analysis (GIVA), Department of Geography and Digital Society Initiative, University of Zurich, Switzerland

**Keywords:** serial memory, route learning, assisted navigation

## Abstract

Primacy and recency features of serial memory are a hallmark of typical memory functions that have been observed for a wide array of tasks. Recently, the ubiquity of this serial position effect has been supported for objects learned during navigation, with canonical serial position functions observed for sequence memory of landmarks that were encountered along a route during a highly controlled virtual navigation task. In the present study, we extended those findings to a real-world navigation task in which participants actively walked a route through a city whilst using a navigation aid featuring either realistic or abstract landmark visualisation styles. Analyses of serial position functions (i.e., absolute sequence knowledge) and sequence lags (i.e., relative sequence knowledge) yielded similar profiles to those observed in a lab based virtual navigation task from previous work and non-spatial list learning studies. There were strong primacy effects for serial position memory in both conditions; recency effects only in the realistic visualisation condition; a non-uniform distribution of item-lags peaking at lag +1; and an overall bias towards positive lags for both visualisation conditions. The findings demonstrate that benchmark serial position memory effects can be observed in uncontrolled, real-world behaviour. In a navigation context, the results support the notion that general memory mechanisms are involved in spatial learning, and that landmark sequence knowledge is a feature of spatial knowledge which is affected by navigation aids.

## 1. Introduction

Learning of items ordered in a sequence has been shown to produce serial position-dependent memory effects across a wide range of tasks and stimuli (Oberauer et al., 2018). In general, items positioned at the beginning and end of a sequence benefit from improved recall and serial position memory compared to items positioned in the middle of a sequence, widely known as primacy and recency effects, respectively. In a recent study, Hilton et al. (2021) demonstrated that bowed serial position curves akin to those observed with traditional list learning tasks, are also present for sequence memory for landmarks that were encountered along a route during a lab based virtual navigation task. This finding supports the ubiquity of primacy and recency effects in memory and provides an insight into the mechanisms of memory involved in spatial navigation. In the present study, we extended those findings to a real-world route navigation scenario with active navigation of participants guided by a navigation aid. We further examined the impact of navigation aid visualisation style on subsequent sequence memory for landmarks encountered along the route and simultaneously displayed on the device.

Serial memory for landmarks encountered during navigation was examined with a virtual navigation task by Hilton et al. (2021). In their study, participants watched passive movement through a virtual building, with navigation decisions being made via keyboard. All aspects of the environment were identical except for landmarks placed at intersections, and participants repeated the route several times. Despite the differences between this navigation task and typical list learning tasks, namely multiple exposures to the landmark sequence, longer retention intervals, and no explicit instruction to learn the sequence, Hilton et al. (2021) found stark serial positions curves with primacy and recency for boundary items, and evidence of relative chaining of landmarks independent of absolute positioning via analysis of item-lags. Item-lag refers to the distance between neighbouring items in the recalled order in terms of the original sequence, with smaller lags showing that participants are more likely to recall items nearby to the one previously placed (Healey et al., 2019).

The presence of serial position dependent memory effects in navigation supports the universality of sequence learning effects and suggests that standard memory mechanisms are recruited during route learning. This is in line with the view that navigation involves many non-specialised cognitive functions (Montello, 2009), likely owing to its fundamental role in survival and daily functioning. Route learning is a good candidate to study the involvement of general cognitive functions in navigation behaviour as it is thought to partially rely on associative memory mechanisms. That is, the development of stimulus-response associations between landmarks and movement directions that enable efficient recollection of specific route details (Waller & Lippa, 2007). Further evidence suggests that the involvement of associative memory extends past isolated landmark-direction pairings by showing that the recall of directional information is primed when landmarks are presented in the same order as previously experienced along a route (as compared to random or reverse presentation orders; Schweizer et al., 1998). Such relationships between neighbouring landmarks and the movements that link them are used by navigators to disambiguate repeated landmarks in an environment and have been referred to as stimulus-response-stimulus associations (Strickrodt et al., 2015). Associative memory functions that are involved in route learning may also extend to the recruitment of serial learning processes for the binding of landmarks to location in memory.

Most experimental studies, including the aforementioned navigation studies, take place in restricted lab settings. However, the call for research to move into more naturalistic, real-world environments is ever-growing (de Sanctis et al., 2021; Park et al., 2018; Shamay-Tsoory & Mendelsohn, 2019). Real world behaviour is influenced by many general uncontrolled factors, but one consideration specific to serial learning is longer and more variable retention intervals between exposure to a sequence and recall, which has been shown to affect the recency aspect of the serial position curve (Glenberg et al., 1983). Overall, establishing the presence of general serial memory effects in a real-world navigation task provides insights into the ubiquity of sequence learning mechanisms and their associated benchmark effects in real-world behaviour, and the general cognitive functions involved in naturalistic spatial learning.

The experiment presented in this paper had participants actively navigate a given route through a city assisted by a mobile-map featuring landmarks that were either presented as abstract representations (non-textured buildings) or realistic representations (textured buildings) of landmarks along the route. Specifically, either the first half of the landmark sequence were realistic, and the latter half were abstract, or vice versa. The main results are presented in Kapaj et al., (2023) which aimed to assess the impact of navigation aid visualisation style on a range of spatial learning measures: navigation efficiency, landmark recognition, route direction knowledge, metric knowledge, and sequence knowledge. The measure of landmark sequence knowledge used a reconstruction of order task with an absolute scoring system whereby items were classified correct if they were placed in the correct position and incorrect for all other placements. The analysis was conducted only in terms of percent correct, with no analyses of serial position effects or other sequence scoring conventions and showed no overall performance difference between realistic and abstract visualisation styles. Kapaj et al. (2023) also found no difference in landmark recognition performance between visualisation styles that may affect serial position memory differences.

In this study, we leverage the sequence data from Kapaj et al., (2023) to examine serial position effects for landmark knowledge in the two landmark visualisation conditions. The goal was to assess whether serial position effects for landmark memory would generalise from a highly controlled virtual navigation task to a more ecologically valid assessment of navigation. Moreover, we investigated whether these serial position effects were affected by the visualisation style of a modern, digital navigation aid. In the present experiment, the real-world environment and procedure differed from previous navigation studies using virtual environments in that it was visually diverse, included uncontrolled distraction, participants physically walked the route, they used a navigation aid, and they experienced the route only once. Based on the findings of Hilton et al. (2021), we predicted that serial position curves for the landmark sequence task would conform to a U shape indicating primacy and recency benefits for the boundary landmarks along the route. For the impact of landmark visualisation style, we expected that primacy or recency effects would be enhanced for the realistic display of those respective landmarks, under the assumption that more realistic landmark display would more effectively guide attention towards those relevant locations thus facilitating encoding (Gardony et al., 2015).

## 2. Method

### 2.1. Participants

There were 46 participants (22 females, mean age: 27.3; 24 males, mean age: 27.8) who participated in exchange for 40 Swiss Francs. The study was approved by the University of Zurich Ethics Committee. Participants provided written informed consent prior to the experiment, reported having normal or corrected to normal vision, and had no history of any physical or psychiatric disorders.

### 2.2. Design

The between-subjects independent variable was landmark visualisation order (realistic first, n = 23; realistic last, n = 23). There were multiple dependent variables including: EEG and eye-tracking, follow-up tasks for landmark recognition, route direction, judgement of relative directions, and landmark free reconstruction of order. The present study only pertains to the data obtained in the landmark free reconstruction of order task. The other measures will be mentioned in the procedure for replicability and clarity, but for more detail and results regarding the other measures, refer to Kapaj et al. (2023).

### 2.3. Route navigation task

**Figure 1:**
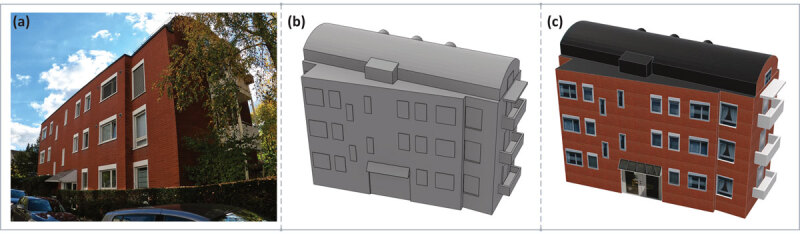
**(a)** Example landmark from the route. **(b)** Example depiction of the landmark in the abstract condition. **(c)** depiction of the landmark in the realistic condition.

In the learning phase, participants navigated a route through the streets of Zurich guided by a mobile-map displayed on a tablet device with a route marked with a black line. The route consisted of 4 left, 5 right and 1 straight movement trajectories at intersections. One building identified as an appropriate landmark from each of the 10 intersections was displayed on the mobile-map. The landmarks were selected via a survey where respondents (n = 9) were provided with photos of the intersections to which they selected the most prominent building, the building they would use when giving directions, and the easiest building to describe. Half the landmarks for each participant were displayed with realistic textures (see Figure 1c) and half were abstract (see Figure 1b).

### 2.4. Procedure

Participants were equipped with EEG and eye-tracking devices in a room close to the route site. Participants were then guided to the route start and instructed to follow the route defined by the mobile-map and to identify the landmarks as they encountered them along the route. Participants were aware that their knowledge of the route would be tested afterwards, but the details of the tasks were not revealed. Participants moved at their own pace and could refer to the mobile-map as often as they liked. If participants made an error when following the route, they were informed by the experimenter and prompted to return to the previous intersection to continue the route. Over all participants only 8 errors were made, and the duration of route navigation was ~10 minutes.

After the route navigation phase, the EEG and eye-tracking devices were removed, and participants returned to a nearby building to complete the follow-up tests. The time from ending the navigation phase to starting the follow-up tests was ~10 minutes and included a trip on public transport. Subsequently, participants completed the landmark recognition task (identifying images of landmarks that were present along the route), the route direction test (indicating the direction of travel at each landmark), the landmark reconstruction of order task, and the judgement of relative direction and distance tests (judging the direction and distance between pairs of landmarks).

The landmark reconstruction of order task involved images of all 10 landmarks on paper (2 randomly chosen colour images per A4 page) being given to participants simultaneously. Participants were asked to write down the position number of each landmark in the sequence on the same paper next to the respective landmark images, based on the order in which they encountered the landmarks along the route. Participants had no time constraints and could amend their responses as often as they liked during the task. The final sequence was recorded after participants indicated they were finished and any ambiguities in the responses were clarified with the participants.

## 3. Results

Analyses were conducted in R (v4.1.1; R Core Team, 2021) using the lme4 package for linear mixed effect models (v1.1-27.1, Bates et al., 2015). For serial position effects, we conducted a generalised linear mixed model (GLMM) on performance (binomial) with the fixed effects of serial position (1–10) with polynomial contrast coding to identify serial position trends, and landmark visualisation order (realistic first or last, sum contrast coding), and the random effect of participant ID (intercept only). We found significant fits for linear (β = –3.64, SE = 0.50, z = –7.28, p < .001) and quadratic (β = 3.01, SE = 0.49, z = 6.22, p < .001) trends for serial position, and no significant fit for a cubic trend (β = 0.26, SE = 0.44, z = 0.60, p = .548). There were also interactions between landmark visualisation order and a linear trend fit (β = –1.28, SE = 0.48, z = –2.68, p = .007) and a cubic fit (β = –1.07, SE = 0.44, z = –2.44, p = .015), but not with a quadratic fit (β = –0.24, SE = 0.47, z = –0.50, p = .615).

To follow up the interactions between serial position trend fit and visualisation order, we repeated the same GLMM separately for each condition. For the realistic first model we found significant fits of both linear (β = –4.81, SE = 0.79, z = –6.07, p < .001) and quadratic trends (β = 2.72, SE = 0.73, z = 3.74, p < .001), but not cubic (β = –0.79, SE = 0.65, z = –1.21, p = .225). For the realistic last model, we found significant fits of linear (β = –2.41, SE = 0.61, z = –3.97, p < .001), quadratic (β = 3.33, SE = 0.64, z = 5.20, p < .001), and cubic (β = 1.37, SE = 0.58, z = 2.34, p = .019) trends. The interaction in the previous model can therefore be explained by the substantially better fit of a linear trend to the serial position data in the realistic first model (compared to a quadratic trend), in contrast to the realistic last model which was best explained with a quadratic trend.

**Figure 2:**
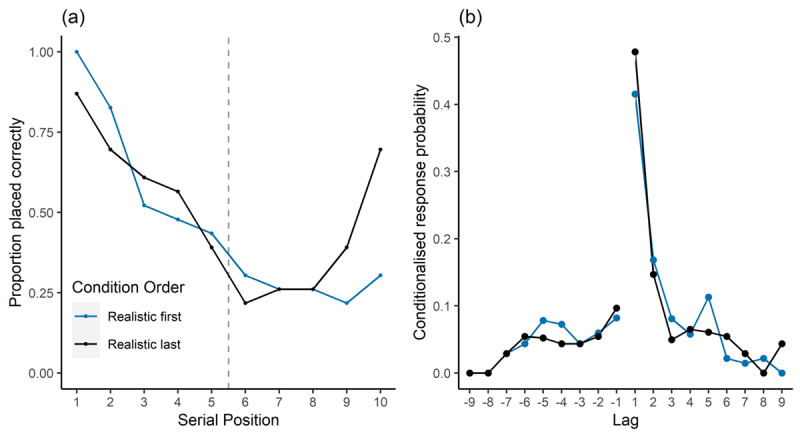
**(a)** Serial position curves revealing strong primacy effects for both conditions, but only recency in the realistic last condition. The dashed line indicates position where landmark visualisation style changed along the route; **(b)** Lag conditionalised response probabilities (lag-CRPs) with a peak at +1, showing forward contiguity effects in reconstruction of landmark order.

Examination of Figure 2a indicated that the differential fits of the two landmark visualisation orders was due to a recency benefit in the realistic last condition that appeared to be absent for the realistic first condition. We confirmed this explanation using post-hoc t-tests with a Bonferroni corrected alpha level of .025, which showed significantly higher performance for the final two landmarks in the realistic last condition (t(43.99) = 2.62, p = .012, *d* = 0.77). In contrast, no significant difference between groups on performance for the first two landmarks was observed (t(33.55) = 1.52, p = .139, *d* = 0.45).

For item-lag we first calculated the distance between each successive pair of landmarks in the given sequences in terms of the original correct order. Possible lags were from –9 to 9 for the 10-item sequence, with negative lags indicating that a landmark was placed *after* an item that it preceded in the original sequence. The number of times each lag of a given value occurred was computed and divided by the number possibilities to make the given lag (e.g., in a 10-item sequence a lag of +1 can occur 10 times, but a lag of +9 can only occur once) to give the item-lag conditionalised response probabilities (lag-CRP). Figure 2b shows the lag-CRPs, with a clear peak at lag +1, indicating good knowledge about the relative ordering of landmarks. There was no significant difference between landmark visualisation orders on lag +1 probabilities (t(43.89) = 0.76, p = .454, *d* = 0.22).

We also analysed the directionality of item-lags using a linear mixed model (LMM) on lag-CRPs with fixed effects of landmark visualisation order (realistic first or last, sum contrast coding) and direction (forward or backward, sum contrast coding) and the random effect of participant ID (intercept only). A main effect of direction showed that forward lags were significantly more likely than backwards lags (β = –0.03, SE < 0.01, t = –11.73, p < .001), with no significant effect of condition (β < 0.01, SE < 0.01, t = 0.01, p = .989) and no significant interaction (β < 0.01, SE < 0.01, t = 0.78, p = .438).^1^

## 4. Discussion

In this study we found that landmark sequence knowledge derived from walking a route through a city guided by a navigation aid broadly conforms to patterns of serial position memory observed in many other contexts. Independent of the landmark visualisation style on the mobile-map display, strong primacy effects were observed for item-position memory, contrary to our hypothesis that primacy would be enhanced in the realistic-first condition. On the other hand, in line with our hypothesis, recency effects were only present when the final landmarks were visualised in a more realistic building style. Lag-CRPs showed strong evidence of relative sequence knowledge for both conditions.

One methodological feature of the present study was the long retention interval between route learning and the reconstruction of order task. In typical sequence learning paradigms, retention intervals are on the scale of <60 seconds, compared to the present retention interval of ~20 minutes including the time to navigate the route and substantial cognitive interference from using public transport to return to the testing location. Glenberg et al. (1983) studied the impact of increased retention intervals and interference on recall serial position effects and they found that primacy was unaffected, but recency was reduced (although not eliminated entirely). In fact, Cortis Mack et al. (2017) found serial position effects for lists presented over very long time intervals (1 item per hour), with clear forward contiguity effects showed by lag-CRPs with similar profiles to those observed in the current study. The results of the current study are in line with these previous lab-based observations of strong primacy effects but weaker recency effects for long retention intervals including potential interference.

Interestingly, the difference between conditions was that recency was greater for the realistic-last condition, despite the retention interval and interference patterns being similar between the two conditions. General visual distinction of latter list items has been shown to increase recency effects in lab-based number sequence learning (Bornstein et al., 1995). The goal of the enhanced landmark visualisation was to improve the direction of attention to the relevant features of the environment by reducing the mismatch between the guidance system and the environment (Richter & Winter, 2014; Wenczel et al., 2017). Our results indicate that landmark-position memory can be enhanced by reducing the visual mismatch between features shown on the mobile-map display and the respective features in the environment, however not for all landmark locations equally. In fact, contrary to our initial expectation of primacy enhancement, the first eight landmarks were unaffected, possibly due to primacy effects reaching ceiling level, which is not unusual (Ward et al., 2010). As a guide for future work aiming to enhance spatial learning from navigation aids, the present study indicates that no one-size-fits-all solution is likely to exist for all route features.

It is possible that we observed only an improvement in recency with the realistic landmark visualisation due to it being the feature of landmark sequence knowledge most impacted by navigation aids. Previous work has shown that multiple facets of spatial learning are negatively affected by navigation aids, such as landmark memory, directional knowledge, or metric knowledge (Dahmani & Bohbot, 2020; Ruginski et al., 2019). However, we are not aware of any studies that have assessed the impact of navigation aids on serial position memory for landmark sequences. Our serial position data for the first 80% of route landmarks is similar to Hilton et al. (2021), where participants navigated without an aid. Hence, it is possible that navigation aid consultation during wayfinding mostly affects recency in landmark sequence knowledge. This could be addressed in future work by contrasting serial position memory for landmarks learned with and without a navigation aid.

A limitation of our study is that landmark identity and serial position were always the same for all participants as a result of real-world experimentation with less control, compared to lab-based studies where items can be randomised across serial positions between subjects. One solution that was overlooked at the time of study design but could be implemented in future work would be to counterbalance the start location and route direction between subjects, so that at least the boundary items along the route may contribute to primacy and recency equally.

Overall, our participants showed sequence knowledge for landmarks that is organised similar to serial memory for non-spatial stimuli. The strong lag-CRP contiguity effect of placing nearby landmarks close to each other shows binding of those items together in memory based on their temporal proximity, alongside their overall position in space as shown by the bowed serial position curves. This study indicates that domain-general serial memory is recruited in real-world active navigation and produces similar effects as in lab-based experiments despite the uncontrolled, noisy nature of a real-world experiment.

## Data Accessibility Statement

The datasets and analysis script from this study are available online at the Open Science Framework open access repository: https://osf.io/jvctb/.

## Footnotes

We repeated this analysis with lags +1 excluded to check if the effects persist in the absence of the large CRP peak at this lag (due to its uniqueness in representing a correct relative ordering of items compared to all other lags which represent an error in ordering) and the patterns of significance were identical to the reported model.
